# Exercise-Induced Ischemic ST-Segment Elevation in Anomalous Origin of the Right Coronary Artery From the Left Sinus of Valsalva With an Intramural Course and Blocked Coronary Bypass

**DOI:** 10.7759/cureus.32418

**Published:** 2022-12-11

**Authors:** Kenta Hirai, Daiki Ousaka, Yosuke Kuroko, Shingo Kasahara

**Affiliations:** 1 Department of Pediatrics, Okayama University School of Medicine, Dentistry, and Pharmaceutical Sciences, Okayama, JPN; 2 Department of Pharmacology, Okayama University School of Medicine, Dentistry, and Pharmaceutical Sciences, Okayama, JPN; 3 Department of Cardiovascular Surgery, Okayama University School of Medicine, Dentistry, and Pharmaceutical Sciences, Okayama, JPN

**Keywords:** sudden cardiac death, treadmill exercise test, diagnostic ct imaging, electrocardiography (ecg), coronary vessel anomalies

## Abstract

Sudden cardiac events in young athletes are a major concern in the field of sports cardiology. Although coronary artery anomalies remain a major cause of cardiac events in young athletes, only a few cases have been diagnosed prior to critical events. Here, we present the case of a previously asymptomatic young male runner who experienced sudden cardiac arrest at the end of a marathon. The patient immediately received cardiopulmonary resuscitation from a bystander and was transported to an emergency hospital. As his electrocardiogram showed ventricular fibrillation, he was treated with electric shock, and his rhythm was successfully converted to a normal sinus rhythm. Following successful resuscitation, the patient was diagnosed with an anomalous origin of the right coronary artery from the left sinus of Valsalva with an intramural course. The patient underwent coronary artery bypass using the right internal thoracic artery. Fifteen years later, the coronary bypass was found to be blocked, but the patient was asymptomatic. However, an exercise electrocardiogram revealed ST-segment elevation in the inferior leads. The patient then underwent an unroofing procedure. He has remained asymptomatic without complications for two years after the second surgery.

## Introduction

Anomalous coronary artery originating from the opposite sinus of Valsalva (ACAOS) is a risk factor for sudden cardiac events (SCEs) in young athletes because of potential myocardial ischemia; however, a few cases are diagnosed prior to critical events [1]. Here, we report the case of an anomalous origin of the right coronary artery from the left sinus of Valsalva with an intramural course (R-ACAOS-IM) in a patient who underwent successful resuscitation after a cardiac arrest that occurred during a marathon. He underwent coronary artery bypass of the right coronary artery (RCA), but the bypass was identified as blocked 15 years later. Although the patient remained asymptomatic despite bypass graft obstruction, an exercise electrocardiogram (ECG) revealed abnormal waveforms of myocardial ischemia that reproduced the premonitory signs of SCE.

## Case presentation

**Figure 1:**
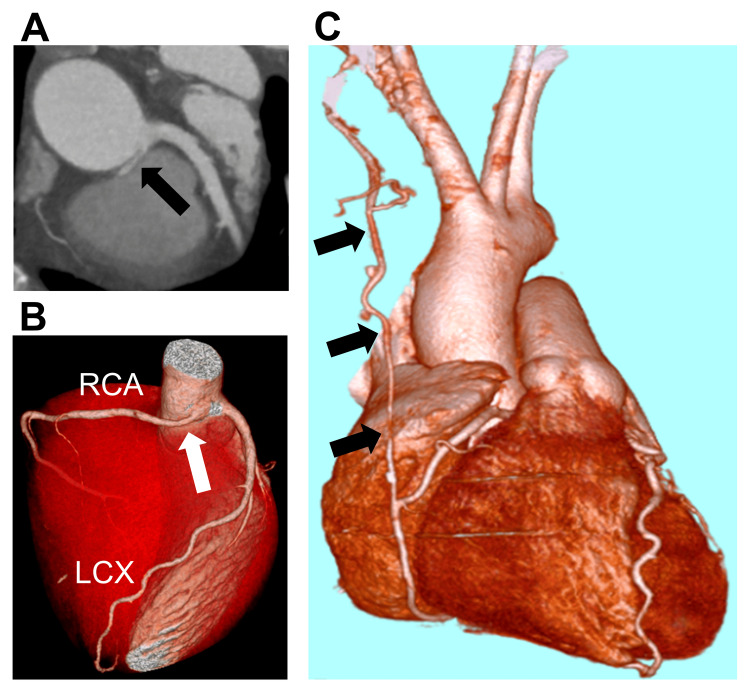
(A) Two- and (B) three-dimensionally enhanced computed tomography scan showing an anomalous origin of the RCA from the left sinus of Valsalva with an intramural course. (C) Bypass of the blocked RCA using the RITA. (A) Black and (B) white arrows indicate the origin of the RCA. (C) Black arrows indicate the bypass graft of the RITA to the RCA. RCA = right coronary artery; RITA = right internal thoracic artery; LCx = left circumflex artery

A 27-year-old Japanese male amateur runner experienced sudden cardiac arrest immediately prior to completing a marathon. He had no history of smoking and no medical or family history of cardiovascular disease or sudden death. He immediately received cardiopulmonary resuscitation from a bystander and was taken to a hospital by ambulance. As the ECG revealed ventricular fibrillation, he received an electric shock in the hospital 15 minutes after the cardiac arrest, and his rhythm was successfully converted to a normal sinus rhythm. He received mechanical ventilation and therapeutic hypothermia for seven days and recovered without any neurological sequelae. He was subsequently transferred to Okayama University Hospital 23 days after the incident to examine the cause of the cardiac arrest. He was diagnosed with R-ACAOS-IM using computed tomography (CT). The RCA originated from the left sinus of Valsalva with an intramural course (Figure 1, Panels A, B). He underwent coronary artery bypass for the RCA using the right internal thoracic artery (RITA) without proximal ligation of the RCA. He was discharged on the sixth postoperative day with preserved cardiac function and no angina. He was treated with aspirin 100 mg once daily and allowed to exercise up to a maximum of 7.5 metabolic equivalents.

**Figure 2:**
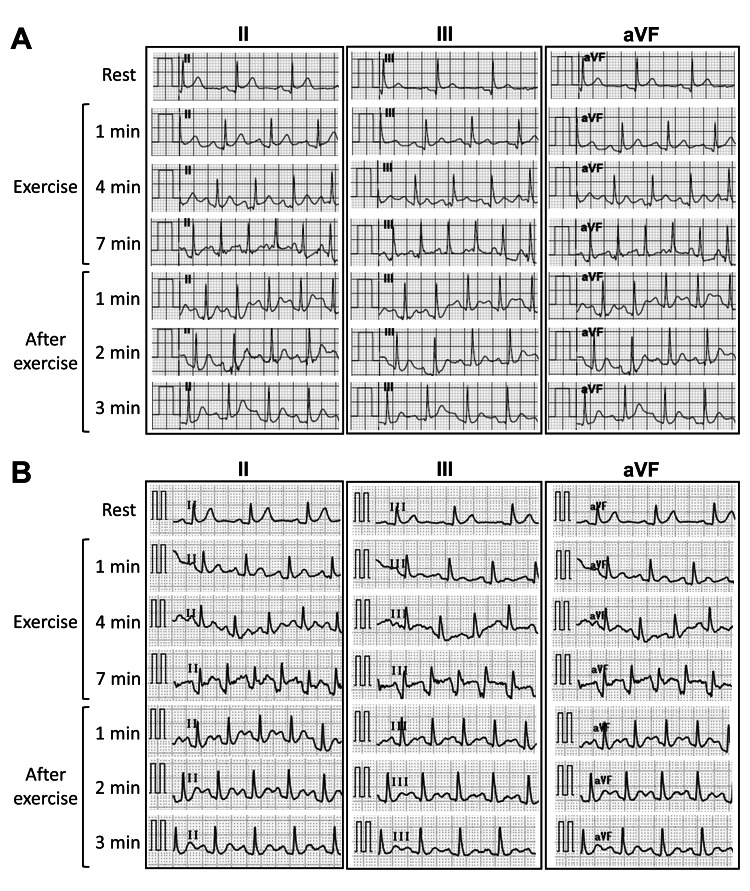
Representative exercise electrocardiograms performed according to Bruce protocol (A) before and (B) after coronary bypass obstruction.

He was followed up every year via CT scans, myocardial scintigraphy, echocardiography, and exercise ECG. Although the RITA-RCA bypass appeared slightly thin on a CT scan taken three years after the bypass surgery, it remained patent for 14 years. No stenosis or calcification of the left coronary artery was observed. Myocardial scintigraphy revealed no signs of ischemia. Preserved cardiac function and wall thickness without asynergy, hyperechoic myocardium, or significant valve regurgitation were observed on echocardiography. An exercise ECG following the Bruce protocol 14 years after the coronary bypass surgery showed no ischemic signs or arrhythmias (Figure 2, Panel A).

When the patient was 42 years old, RITA-RCA bypass obstruction was confirmed on routine CT (Figure 1, Panel C). He remained asymptomatic for 15 years after the bypass surgery. Echocardiography showed preserved cardiac function without asynergy despite the bypass graft obstruction. The patient underwent an exercise ECG following the Bruce protocol to assess the need for reoperation. In the resting condition, slight ST-segment elevations revealed suspected early repolarization in leads II, III, and aVF. ST-segment elevations in leads II, III, and aVF were observed seven minutes after exercise (Figure 2, Panel B). The waveforms suggested inferior myocardial ischemia in response to obstruction of the RCA origin triggered by exercise. Although the patient remained asymptomatic even when the ST-segment elevation appeared, we stopped him from running, and the ST-segment elevation immediately improved. The patient underwent an unroofing procedure for R-ACAOS-IM at our hospital, and the blocked RITA-RCA bypass was ligated. One year after the unroofing procedure, exercise ECG according to the Bruce protocol showed no ischemic signs or arrhythmias. He has remained asymptomatic and free of complications for two years following the second surgery.

## Discussion

SCE associated with physical activity is rare; however, it can significantly impact a patient’s social life. The recent increase in participation in sports such as marathons has made SCE a major concern for both athletes and the general population. One study reported an incidence of cardiac arrest of 0.54 per 100,000 participants in long-distance races, the main causes of which were hypertrophic cardiomyopathy and myocardial ischemia [2]. The annual incidence of sudden cardiac death among young athletes (age <35 years) was reportedly 0.7-3.0 per 100,000 athletes [3]. Of the 1,049 patients aged 8-39 years with SCEs, 677 (65%) were ≤17 years of age [4]. While the most common cause of SCEs in older athletes is degenerative coronary artery disease, congenital or inherited cardiac abnormalities are the primary risk factors in younger athletes.

A previous report showed that 39 of 126 cases (31%) of non-traumatic sudden death among young athletes were caused by coronary artery anomalies [5]. Similarly, one autopsy study showed that 38-66% of ACAOS cases were asymptomatic before death and 75-83% died during exercise [6]. Previous case reports suggested that SCEs in young runners can be attributed to R-ACAOS-IM [7,8]. Typical acute waveforms of ischemic events in R-ACAOS-IM patients are indicated as ST-segment elevations of up to 0.2 mV in leads II, III, and aVF, and subsequent waveforms as normalized ST-segment elevations and negative T-waves in leads II, III, and aVF [7]. While unroofing of an anomalous coronary artery is performed safely in most patients, coronary artery bypass is an alternative option for patients with concomitant atherosclerotic coronary artery disease or emergency cases of myocardial ischemia [9-11]. However, a retrospective analysis identified that two patients who previously underwent RITA-RCA bypass without proximal ligation of the native coronary artery experienced recurrent angina with blocked RITAs five to seven months after bypass grafting [12]. Because the flow in an anomalous vessel is typically compromised only during periods of stress or exercise, there is usually substantial competitive flow between the RITA and RCA, which can cause a bypass obstruction [10,12].

In the present case, although the unroofing procedure was also considered as the first surgery, we decided to perform a coronary bypass without proximal ligation of the native coronary artery because of the lack of accumulated knowledge. We performed an unroofing procedure as the second surgery according to studies published after the first surgery [10,12]. We recently performed an unroofing procedure as the first surgery for other patients with ACAOS-IM.

## Conclusions

The patient described here experienced cardiac arrest during a marathon and underwent RITA-RCA bypass surgery after successful resuscitation. Fifteen years later, the bypass became blocked and RCA perfusion was limited by the narrow RCA origin, which was the same as the preoperative hemodynamics. An exercise ECG following coronary bypass obstruction indicated typical waveforms of inferior myocardial ischemia among patients with R-ACAOS-IM. Regular examination of exercise ECG and coronary artery imaging is important for patients with R-ACAOS-IM with RITA-RCA bypass for detecting signs of obstruction and exertional angina pectoris.
